# External Validation of an Upgraded AI Model for Screening Ileocolic Intussusception Using Pediatric Abdominal Radiographs: Multicenter Retrospective Study

**DOI:** 10.2196/72097

**Published:** 2025-07-08

**Authors:** Jeong Hoon Lee, Pyeong Hwa Kim, Nak-Hoon Son, Kyunghwa Han, Yeseul Kang, Sejin Jeong, Eun-Kyung Kim, Haesung Yoon, Sergios Gatidis, Shreyas Vasanawala, Hee Mang Yoon, Hyun Joo Shin

**Affiliations:** 1 Department of Radiology, Stanford Medicine, Stanford, CA, United States; 2 Department of Radiology and Research Institute of Radiology, University of Ulsan College of Medicine, Asan Medical Center, Seoul, Republic of Korea; 3 Department of Statistics, Keimyung University, Daegu, Republic of Korea; 4 Department of Radiology, Research Institute of Radiological Science and Center for Clinical Imaging Data Science, Yonsei University College of Medicine, Severance Hospital, Seoul, Republic of Korea; 5 Department of Radiology, Research Institute of Radiological Science and Center for Clinical Imaging Data Science, Yonsei University College of Medicine, Yongin Severance Hospital, 363, Dongbaekjukjeon-daero, Giheung-gu, Yongin-si, Gyeonggi-do, 16995, Republic of Korea, 82 31 5189 8364; 6 Department of Radiology, Research Institute of Radiological Science and Center for Clinical Imaging Data Science, Yonsei University College of Medicine, Gangnam Severance Hospital, Seoul, Republic of Korea

**Keywords:** intussusception, artificial intelligence, abdominal radiography, ultrasonography, radiologists

## Abstract

**Background:**

Artificial intelligence (AI) is increasingly used in radiology, but its development in pediatric imaging remains limited, particularly for emergent conditions. Ileocolic intussusception is an important cause of acute abdominal pain in infants and toddlers and requires timely diagnosis to prevent complications such as bowel ischemia or perforation. While ultrasonography is the diagnostic standard due to its high sensitivity and specificity, its accessibility may be limited, especially outside tertiary centers. Abdominal radiographs (AXRs), despite their limited sensitivity, are often the first-line imaging modality in clinical practice. In this context, AI could support early screening and triage by analyzing AXRs and identifying patients who require further ultrasonography evaluation.

**Objective:**

This study aimed to upgrade and externally validate an AI model for screening ileocolic intussusception using pediatric AXRs with multicenter data and to assess the diagnostic performance of the model in comparison with radiologists of varying experience levels with and without AI assistance.

**Methods:**

This retrospective study included pediatric patients (≤5 years) who underwent both AXRs and ultrasonography for suspected intussusception. Based on the preliminary study from hospital A, the AI model was retrained using data from hospital B and validated with external datasets from hospitals C and D. Diagnostic performance of the upgraded AI model was evaluated using sensitivity, specificity, and the area under the receiver operating characteristic curve (AUC). A reader study was conducted with 3 radiologists, including 2 trainees and 1 pediatric radiologist, to evaluate diagnostic performance with and without AI assistance.

**Results:**

Based on the previously developed AI model trained on 746 patients from hospital A, an additional 431 patients from hospital B (including 143 intussusception cases) were used for further training to develop an upgraded AI model. External validation was conducted using data from hospital C (n=68; 19 intussusception cases) and hospital D (n=90; 30 intussusception cases). The upgraded AI model achieved a sensitivity of 81.7% (95% CI 68.6%‐90%) and a specificity of 81.7% (95% CI 73.3%‐87.8%), with an AUC of 86.2% (95% CI 79.2%‐92.1%) in the external validation set. Without AI assistance, radiologists showed lower performance (overall AUC 64%; sensitivity 49.7%; specificity 77.1%). With AI assistance, radiologists’ specificity improved to 93% (difference +15.9%; *P*<.001), and AUC increased to 79.2% (difference +15.2%; *P*=.05). The least experienced reader showed the largest improvement in specificity (+37.6%; *P*<.001) and AUC (+14.7%; *P*=.08).

**Conclusions:**

The upgraded AI model improved diagnostic performance for screening ileocolic intussusception on pediatric AXRs. It effectively enhanced the specificity and overall accuracy of radiologists, particularly those with less experience in pediatric radiology. A user-friendly software platform was introduced to support broader clinical validation and underscores the potential of AI as a screening and triage tool in pediatric emergency settings.

## Introduction

Intussusception is an important abdominal emergency in young infants caused by the invagination of a bowel segment into the proximal lumen, potentially leading to obstruction, ischemia, and fatal complications such as perforation and peritonitis, if untreated [1]. The classic clinical triad of cyclic irritability, currant jelly stool, and a palpable abdominal mass is observed in fewer than 50% of cases, making diagnosis based solely on clinical presentation challenging [1,2]. Infants’ inability to accurately articulate symptoms and the difficulty of physical examination further emphasize the critical role of imaging in diagnosis.

Abdominal radiography is the initial imaging modality for children with abdominal symptoms. Although specific signs such as the target or meniscus sign in the right upper quadrant may suggest intussusception, the sensitivity of abdominal radiographs (AXRs) remains low, ranging from 45% to 60% [2]. Nevertheless, radiographs are essential for evaluating bowel gas patterns, excluding alternative diagnoses, and identifying pneumoperitoneum prior to therapeutic reduction. For definitive diagnosis, abdominal ultrasonography is the modality of choice, offering sensitivity and specificity exceeding 97% by detecting the characteristic target or doughnut-shaped bowel loops [1,3]. Despite its accuracy, ultrasonography availability is limited in many hospitals lacking 24-hour radiologists or radiographers, delaying diagnosis and increasing the risk of bowel obstruction progression. Conversely, hospitals with 24-hour access may experience a high volume of unnecessary ultrasonography requests for patients with nonspecific symptoms, contributing to increased workload and health care costs.

Recently, artificial intelligence (AI) has emerged as a promising tool for radiologists, particularly in the analysis of medical images for disease screening or diagnosis [4]. While its application in pediatric abdominal radiology remains limited, a few attempts have been made to implement AI for pediatric abdominal emergency diseases, including the detection of intussusception [5,6]. While ultrasonography already provides high diagnostic performance for intussusception when performed, recent studies have explored the use of AI to directly analyze ultrasonography images for detection [3,7,8]. However, the critical challenge lies in determining which patients should undergo ultrasonography in the first place. Using AI for this triage process could optimize workflows, reduce unnecessary examinations, and prevent diagnostic delays. In 2019, a study first proposed the use of AI to analyze AXRs for identifying pediatric patients requiring ultrasonography for intussusception diagnosis [6]. Although a subsequent validation study using a similar approach was conducted, no further advancements or applications of this concept have been reported since 2020 [9]. Meanwhile, advancements in AI technology have continued, highlighting the need for external validation studies using multicenter data to evaluate the clinical utility of updated algorithms.

Therefore, the purpose of this study was to develop an upgraded AI model and validate it using multicenter data for the screening of ileocolic intussusception on pediatric AXRs. Additionally, the study aimed to present a software platform for practical clinical application. We hypothesized that the upgraded AI model would maintain reasonable diagnostic performance across external multicenter datasets and could potentially assist in the screening of pediatric emergency abdominal conditions that require further evaluation.

## Methods

### Ethical Considerations

The Institutional Review Board of Yongin Severance Hospital (protocol 9-2022-0102) approved this multicenter retrospective study and waived the requirement for informed consent. This research was conducted in accordance with the Declaration of Helsinki and adhered to the STROBE (Strengthening the Reporting of Observational Studies in Epidemiology) guidelines. All data used in this study were fully anonymized prior to analysis, and no identifiable personal information was collected or retained. This study began with an AI model developed from data at hospital A during a preliminary study [6], which was then upgraded by incorporating additional data from hospital B. The upgraded algorithm was externally validated using data from hospitals C and D.

### Participants

**Figure 1.**
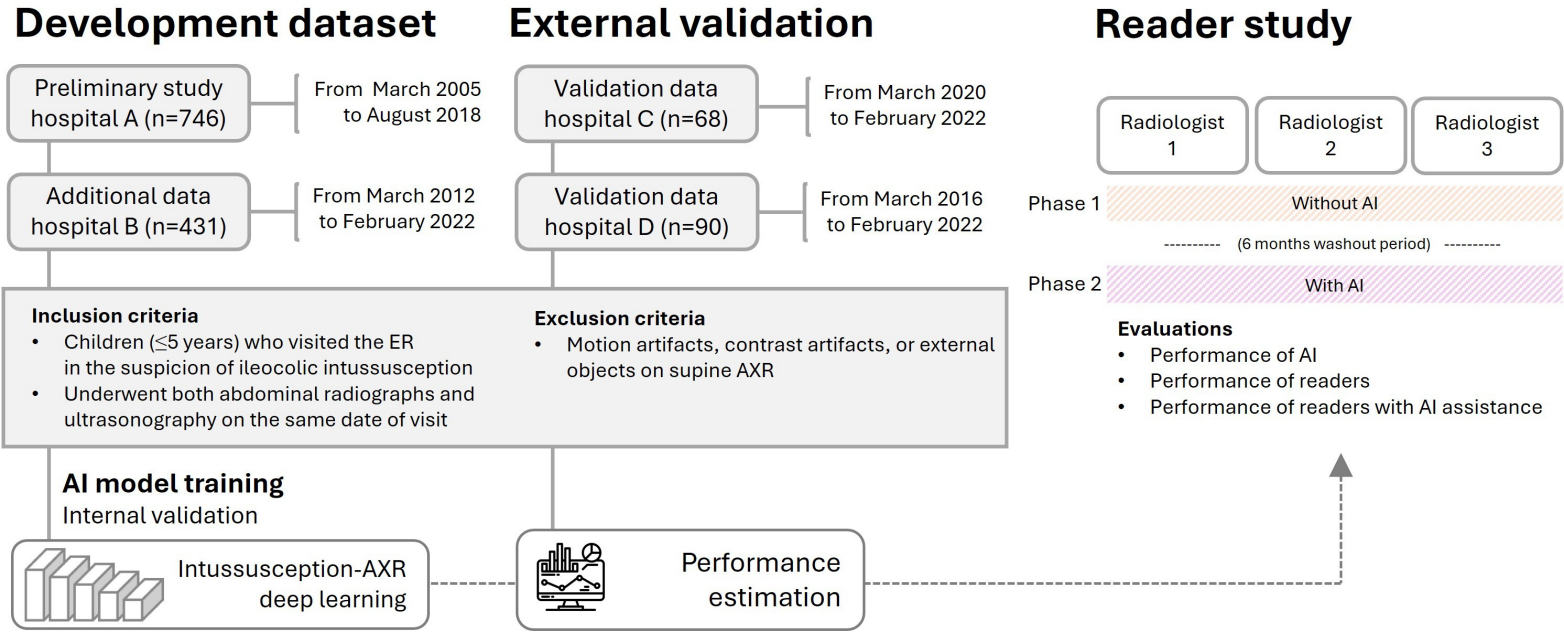
Study workflow for predicting ileocolic intussusception in pediatric patients using AXRs. AI: artificial intelligence; AXR: abdominal radiograph; ER: emergency department.

Pediatric patients (≤5 years) who visited the emergency department and underwent both AXRs and ultrasonography on the same date of visit for suspected ileocolic intussusception were included retrospectively, following the same inclusion criteria established in the preliminary study at hospital A [6]. Data were collected from March 2012 to February 2022 at hospital B, from March 2020 to February 2022 at hospital C, and from March 2016 to February 2022 at hospital D. We included patients who had AXRs before the reduction of intussusception. Patients with motion artifacts, contrast artifacts, or external objects such as buttons or metallic accessories on their abdominal supine radiographs were excluded. The flowchart of patient inclusion and exclusion is presented in Figure 1.

Patients were categorized into the control and intussusception groups based on the ultrasonography results. Due to the inevitable numerical imbalance between the 2 groups in real-world clinical settings, where children without intussusception are more prevalent, we included all children with intussusception during the study period in the intussusception group. For the control group, we included 3 to 4 times the number of patients compared to the intussusception group, including cases in chronological order starting from the most recent date [6].

Regarding the AXRs, we included only the initial radiographs in the supine position, as intussusception is most common in infants and young children who are often unable to stand unassisted. While the supine view is routinely performed for this age group, upright views are obtained selectively, so only the standard supine views were included in the analysis. Digital Imaging and Communications in Medicine format images were saved using the picture archiving and communication system.

Collected data from 4 hospitals (A: n=746, B: n=431, C: n=68, and D: n=90) included children (≤5 years) who visited the emergency department and underwent both supine AXRs and ultrasonography due to suspected intussusception. Cases with artifacts or external objects on AXR were excluded. The intussusception-AXR deep learning model was trained using data from hospitals A and B and externally validated with data from hospitals C and D. In the reader study, radiologists evaluated AXR with and without AI assistance in a 2-phase design, separated by a washout period. The evaluations assessed the performance of readers, the performance of readers with AI assistance, and the performance of AI alone.

### AI Model Development

We developed a deep learning model using data from hospitals A and B as the development set, with 10% of the data randomly selected for the internal test dataset. To further assess the model’s robustness, we performed 5-fold cross-validation on the remaining 90% training data, selecting the epoch with the highest area under the receiver operating characteristic curve (AUC) for each validation fold. Each of the 5 resulting models was then evaluated on the internal test set. The Medical Image Processing, Analysis, and Visualization Software (version 8.0.2; Center for Information Technology, National Institutes of Health) was used to draw rectangular regions of interest (ROIs), consistent with those in the previous study [6]. Each radiograph was annotated to indicate whether intussusception was present or not, based on abdominal ultrasonography results. The ROIs were drawn by a single board-certified pediatric radiologist with more than 10 years of experience. The rectangular ROIs were placed to cover the right abdomen from the right diaphragm to the right iliac crest, lateral to the vertebral bodies, based on consistent anatomical landmarks, as described in the previous study. Given the standardized anatomical boundaries, the potential for significant interannotator variability was considered minimal.

The input images were preprocessed to 380×380 pixels with 3 channels. We performed extensive data augmentation to improve model generalization, including (1) random ROI-based cropping with varying positions relative to the annotated lesions; (2) horizontal and vertical flips; (3) random rotations within ±30 degrees; (4) color jittering with random adjustments to brightness, contrast, saturation, and hue; and (5) *z* score normalization applied independently to each channel.

We implemented an EfficientNet-B4 architecture–based classification model [10]. The network processes the augmented images through multiple convolutional blocks, followed by 2 fully connected layers for final binary classification. The model was trained using cross-entropy loss and optimized with an AdamW optimizer. The learning rate was adjusted using a cosine annealing schedule, which cyclically varies the learning rate between the initial value and 0 following a cosine curve over each training cycle. The optimal model weights were selected based on the lowest loss achieved on the internal test dataset and were subsequently used for evaluation on external validation datasets.

**Figure 2.**
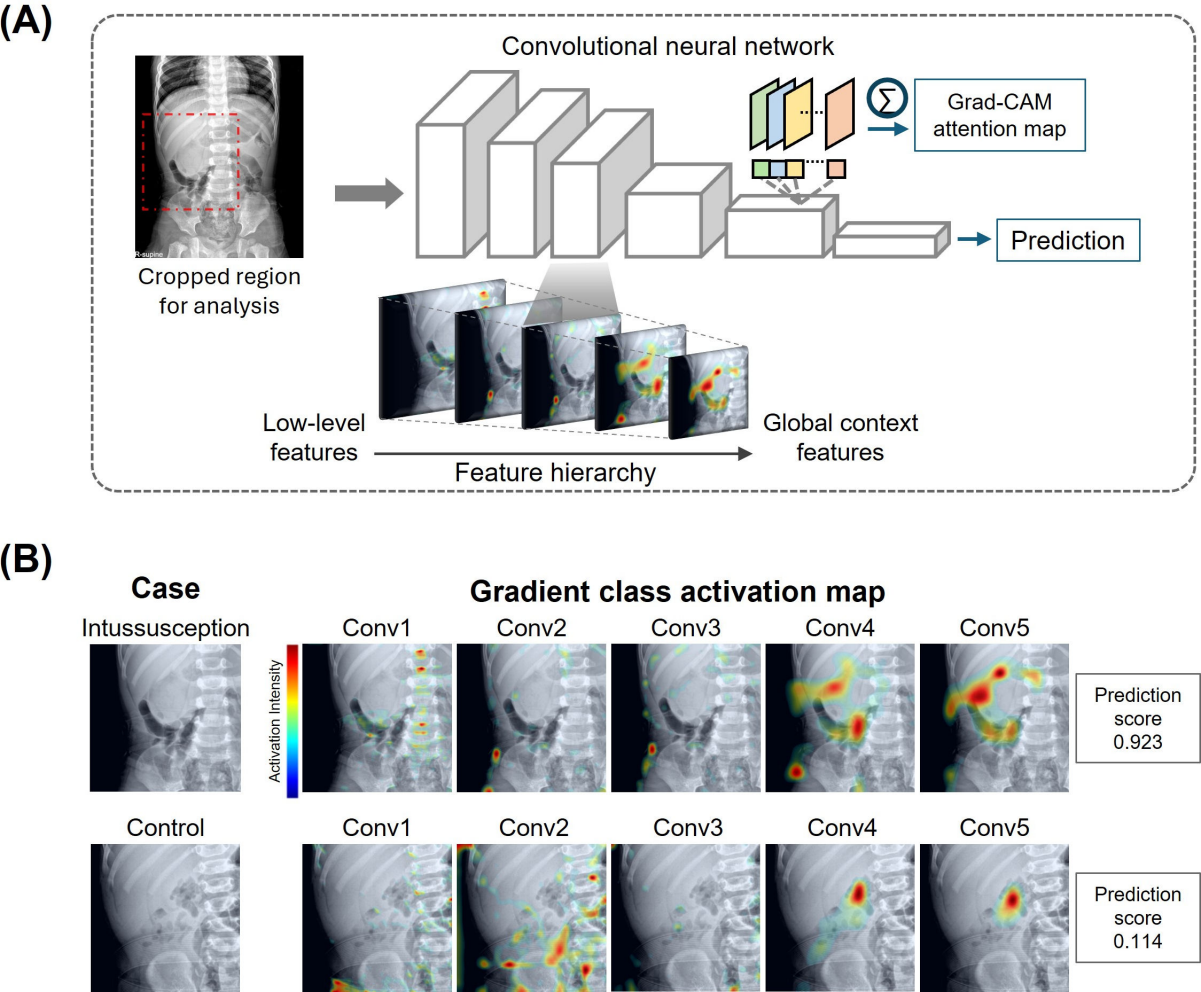
Overview of the artificial intelligence model architecture and visualization of model interpretability. (A) Schematic diagram of the deep learning model architecture showing the progression from input image through convolutional layers to final prediction, including Grad-CAM attention map generation. (B) Representative cases demonstrating Grad-CAM visualizations across different convolutional layers for both intussusception and normal cases. Conv: convolution; Grad-CAM: Gradient-Weighted Class Activation Mapping.

To interpret the model’s decision-making process, we used Gradient-Weighted Class Activation Mapping (Grad-CAM) to generate visual explanations highlighting the regions most influential in classification across 5 different network layers. This visualization approach provides insights into the model’s attention to specific anatomical areas when making predictions (Figure 2).

### External Validation of AI With Reader Study

For external validation, data from hospitals C and D were used. Three radiologists independently assessed the presence of ileocolic intussusception using AXRs from these hospitals. The readers included 2 radiology residents (first year [radiologist 1] and second year [radiologist 2]) and 1 board-certified pediatric radiologist with more than 10 years of specialized experience (radiologist 3). Radiologists were selected to represent different levels of clinical experience, allowing evaluation of how AI assistance might affect diagnostic performance across varying expertise levels. This retrospective reader study design aimed to simulate real-world clinical practice and serve as a foundational investigation for future prospective validation studies. Before the independent evaluation phase, the radiologists participated in a short calibration session by jointly reviewing a small number of sample cases to standardize basic interpretation criteria. Subsequently, all assessments were conducted independently under blinded and randomized conditions. For each radiograph interpretation, radiologists were shown both intussusception and control class Grad-CAM visualizations from the final convolutional block along with their respective prediction scores, highlighting the regions most influential in the AI model’s decision-making for each case. After a 6-month washout period, the same radiographs were re-evaluated by the 3 radiologists with access to the upgraded AI analysis results to determine the presence of intussusception. Diagnostic performance was compared between the radiologists and the upgraded AI algorithm. Additionally, the performances of the radiologists were analyzed within each stage, comparing their assessments without and with AI assistance.

### Statistical Analysis

Statistical analyses were performed using Python (version 3.12.5; Python Software Foundation) with the *statsmodels, scikit-learn*, and *numpy* libraries. Diagnostic performance was assessed using sensitivity, specificity, and AUC, with 95% CI. The performance of each individual reader and the overall reader performance (ie, with AI assistance and without AI assistance) were evaluated and compared with those of the AI model. Sensitivity and specificity were calculated using true positives, false negatives, true negatives, and false positives, and their 95% CIs were estimated using the Clopper-Pearson method. AUC values and their 95% CIs were calculated using bootstrapping with 1000 resamples. For the 5-fold cross-validation analysis, AUC and CIs were estimated using the DeLong method. Comparisons of diagnostic performance included (1) performance differences between AI and each reader with and without AI assistance, (2) comparisons between AI and the overall reader performance (with AI and without AI assistance), and (3) within-reader comparisons of performance with and without AI assistance. Sensitivity and specificity differences were assessed using logistic regression with generalized estimating equations, which accounted for the correlation of repeated measurements within the same dataset. AUC differences were evaluated using the DeLong test, a nonparametric method for assessing the statistical significance of differences in AUC values. Statistical significance was defined as a *P* value less than .05.

## Results

### Participants

During the study period, 431 patients (male: n=265; female: n=166; mean age 2, SD 1.7 years) with 143 cases of intussusception were included at hospital B. The radiographs of these patients were used for additional training to develop an upgraded AI model based on the preliminary study, which included a total of 746 patients, including 246 cases of intussusception, from hospital A. For validation of the algorithm, 68 patients (male: n=42; female: n=26; mean age 1.6, SD 1.5 years) with 19 cases of intussusception from hospital C and 90 patients (male: n=47; female: n=43; mean age 1.5, SD 1.3 years) with 30 cases of intussusception from hospital D were included (Figure 1).

### Diagnostic Performance of the Upgraded AI Model

**Figure 3.**
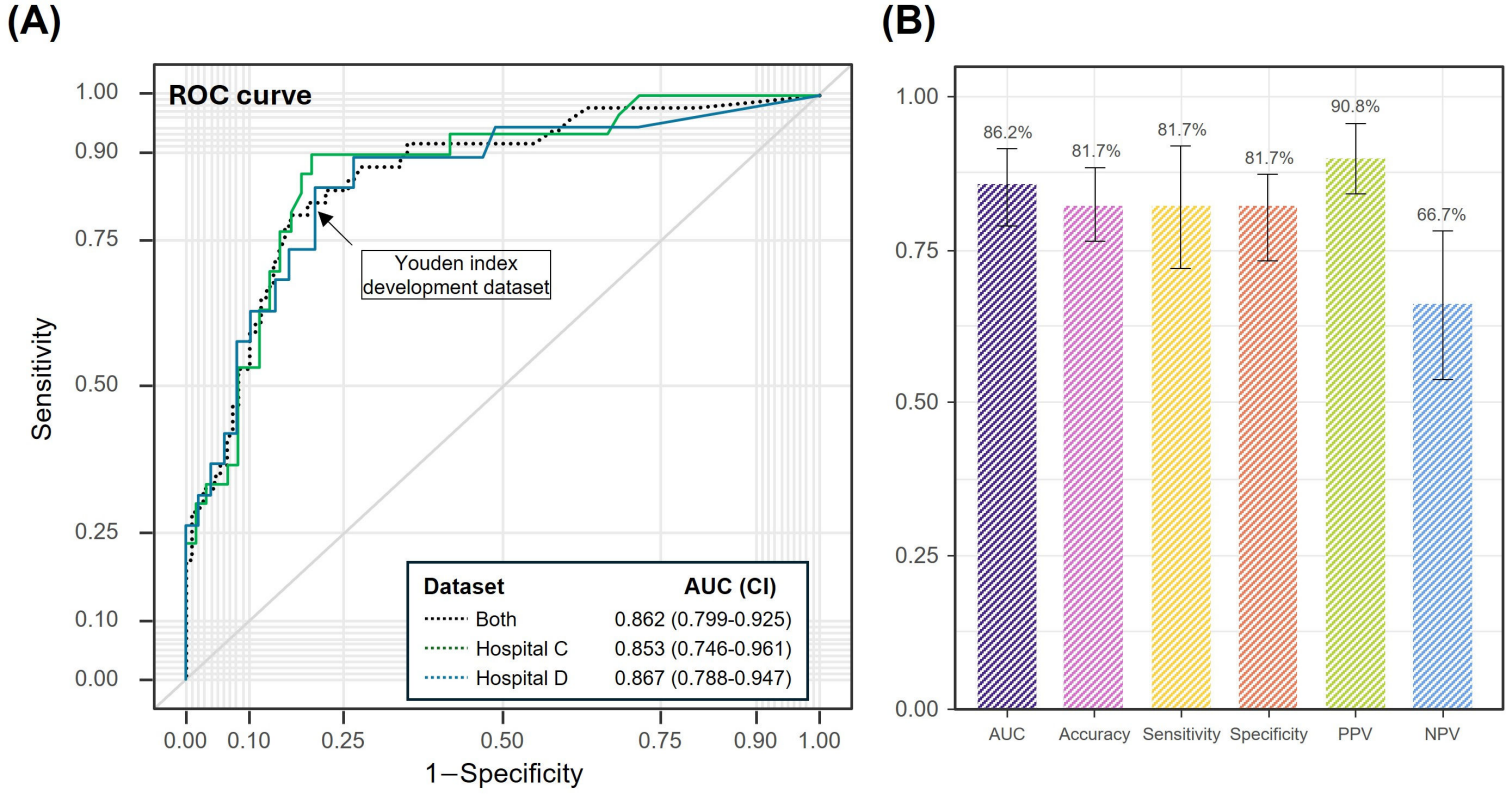
Diagnostic performance of the AI model on external validation datasets. (A) ROC curves showing performance on hospital C, hospital D, and combined datasets. (B) Overall diagnostic metrics, including AUC, accuracy, sensitivity, specificity, PPV, and NPV with 95% CIs. AUC: area under the receiver operating characteristic curve; NPV: negative predictive value; PPV: positive predictive value; ROC: receiver operating characteristic.

Using external validation datasets from hospital C (n=68) and hospital D (n=90), our model demonstrated an overall AUC of 86.2% (95% CI 79.2%‐92.1%; Figure 3). Based on the optimal classification threshold of 0.3035, determined by the Youden index from the internal test dataset, our model achieved consistent performance across both external cohorts with accuracy, sensitivity, and specificity all at 81.7%. The positive predictive value and negative predictive value were 90.8% and 66.7%, respectively (Table 1). When analyzed separately, the model achieved AUC values of 0.853 (95% CI 74.6%‐96.1%) for hospital C and 86.7% (95% CI 78.8%‐94.7%) for hospital D. The performance of individual readers with and without AI assistance is summarized in Multimedia Appendix 1, along with the receiver operating characteristic curve of the AI model.

**Table 1.** Diagnostic performance of artificial intelligence (AI) and readers.

Readers and condition		Sensitivity (95% CI)	*P* value (versus AI)	Sensitivity change	*P* value	Specificity (95% CI)	*P* value (versus AI)	Specificity change	*P* value	AUC^a^ (95% CI)^b^	*P* value (versus AI)^b^	AUC change^b^	*P* value
AI		0.817 (0.686‐0.900)	N/A^c^	N/A	N/A	0.817 (0.733‐0.878)	N/A	N/A	N/A	0.862 (0.792‐0.921)	N/A	N/A	N/A
Radiologist 1				−0.082	.37			+0.376	<.001			+0.147	.08
	Without AI	0.592 (0.452‐0.718)	.02			0.550 (0.457‐0.641)	<.001			0.571 (0.485‐0.655)	<.001		
	With AI	0.510 (0.374‐0.645)	<.001			0.927 (0.857‐0.962)	<.001			0.718 (0.644‐0.795)	.005		
Radiologist 2				−0.02	.76			+0.055	.08			+0.017	.83
	Without AI	0.429 (0.300‐0.568)	<.001			0.872 (0.796‐0.922)	.19			0.650 (0.569‐0.729)	<.001		
	With AI	0.408 (0.300‐0.548)	<.001			0.927 (0.796‐0.962)	.01			0.667 (0.590‐0.738)	<.001		
Radiologist 3				+0.041	.48			+0.046	.13			+0.043	.58
	Without AI	0.469 (0.337‐0.607)	<.001			0.890 (0.817‐0.936)	.10			0.680 (0.601‐0.758)	<.001		
	With AI	0.510 (0.337‐0.645)	<.001			0.936 (0.817‐0.968)	<.001			0.723 (0.648‐0.796)	.01		
Overall radiologists				−0.021	.37			+0.159	<.001			+0.152	.05
	Without AI	0.497 (0.417‐0.577)	<.001			0.771 (0.722‐0.813)	<.001			0.640 (0.553‐0.713)	<.001		
	With AI	0.476 (0.397‐0.557)	<.001			0.930 (0.897‐0.953)	.01			0.792 (0.723‐0.866)	.15		

a AUC: area under the receiver operating characteristic curve.

b DeLong test for AUC; all other analyses were performed using logistic regression with the generalized estimating equation. AUC and CIs were estimated using 1000 bootstrap resamples.

c N/A: not applicable.

To assess the model’s robustness, we performed 5-fold cross-validation on the training data and evaluated each fold-specific model on the fixed internal test set and both external validation cohorts. The mean AUC across the 5 folds was 0.928 on the internal test set and 0.843 on the combined external cohorts (Multimedia Appendix 2 ).

### External Validation of AI With Reader Study

Table 1 summarizes the diagnostic performance of individual radiologists and compares their results with the AI model. Without AI assistance, radiologists exhibited significantly lower overall sensitivity than AI (49.7% vs 81.7%; *P*<.001). Overall specificity was also significantly lower in radiologists compared to AI (77.1% vs 81.7%; *P*<.001). However, when analyzing individual performance, radiologist 2 and radiologist 3 had higher specificity (87.2% and 89%, respectively) than AI, though the differences were not statistically significant. In contrast, radiologist 1 had a significantly lower specificity compared to AI (55%; *P*<.001).

With AI assistance, overall sensitivity remained significantly lower for radiologists compared to AI (47.6% vs 81.7%; *P*<.001). However, sensitivity improved slightly for radiologist 3 (51%), while radiologist 1 and radiologist 2 showed minor decreases (51% and 40.8%, respectively). These changes were not statistically significant when compared to their performance without AI. Notably, overall specificity improved significantly with AI assistance (93% vs 81.7%; *P*=.01), marking a significant increase from the results without AI (+15.9%; *P*<.001). The most notable improvement in specificity was observed in radiologist 1, whose specificity increased by 37.6% (*P*<.001), reflecting the greatest gain among the 3 radiologists, as radiologist 1 initially had the lowest specificity without AI.

Regarding AUC performance, the overall AUC of radiologists without AI assistance was significantly lower than that of AI (64% vs 86.2%; *P*<.001). With AI assistance, the overall AUC improved to 79.2%, reaching a level not significantly different from AI alone (*P*=.15). The overall AUC increase was +15.2% (*P*=.05), with the greatest improvement observed in radiologist 1.

## Discussion

### Principal Findings

This study presents an upgraded AI model, which demonstrated both sensitivity and specificity of 81.7% for detecting ileocolic intussusception on AXRs of young children. The AI model outperformed radiologists without AI assistance. With AI assistance, the specificity of radiologists improved to 93%, which was significantly higher than that of AI alone. Additionally, AI assistance led to a 15.2% increase in the overall AUC for radiologists in detecting intussusception on radiographs, highlighting its potential role as a supportive tool for screening ileocolic intussusception in pediatric imaging. The system is freely available on the web [11].

Nowadays, AI has become increasingly integrated into the daily practice of radiologists, especially in adult imaging. Commercial AI software has emerged and been integrated into hospital-wide settings, demonstrating the advantages of its use in daily practice [12]. When focusing on radiographs, recent studies have suggested that AI could enhance the diagnostic performance of emergent or life-threatening diseases, such as pneumothorax and lung cancer on chest radiographs [13-17]. Similar improvements have been observed in musculoskeletal radiographs, including bone age assessment and fracture detection [18-23]. This advancement could help reduce the workload of radiologists by decreasing reading time and alleviating concerns about missing cases [24-26]. However, in the era of pediatric radiology, the adoption of AI is just beginning, primarily for research purposes, as it is challenging to obtain qualified, labeled, sufficient, and large datasets specific to pediatric disease entities in growing children [4,6,27]. Although there have been some efforts to demonstrate the benefits of using AI for pediatric chest radiographs, studies on AXRs applying AI to pediatric-specific or emergent diseases remain scarce [28-31].

### Comparison to Prior Work

In 2019, the authors first demonstrated the potential use of AI on pediatric AXRs for screening ileocolic intussusception [6]. Given that intussusception is a crucial emergent abdominal disease in young infants requiring immediate treatment, they sought to determine whether AI could aid in identifying children needing further ultrasonography evaluation using AXRs. This first AI algorithm exhibited superior sensitivity (76% vs 46%; *P*=.01) and showed no significantly different specificity (96% vs 92%; *P*=.32) compared to radiologists in detecting intussusception on radiographs [6]. This was significant, as it marked the first application of AI on pediatric AXRs and in intussusception. Subsequently, another group validated this attempt with their patient data, yielding similar results [9]. To date, there have been few attempts to use AI on ultrasonography to detect intussusception. However, since ultrasonography detection of intussusception has high diagnostic performance even with trainees, the clinical benefits of using AI could be more emphasized on the initial screening side using radiographs, rather than ultrasonography, to determine which patients need further work-up with ultrasonography at the time of acquiring AXRs [3,8]. Therefore, this study aimed to upgrade the AI model in line with technological advancements since 2019, validate it using multicenter data, and introduce a practical software platform.

In this study, the results were generally consistent with previous studies but demonstrated improved performance, particularly in sensitivity, compared to the previous AI model (81.7% vs 76%). Additionally, the AI model outperformed radiologists overall, with a higher AUC (86.2% vs 64%). Regarding the low sensitivity, the sensitivity of radiologists for detecting intussusception using AXRs varies from 45% to 79% in the literature [1,2], and the diagnostic performance of radiologists in this study showed a similar trend [2], highlighting the effectiveness of AI as a screening tool for intussusception. The lack of a significant change in sensitivity after using AI may raise questions about its effectiveness. However, considering that radiologists’ conventional diagnostic performance for detecting intussusception on AXRs is inherently lower than that of ultrasonography, this result is not unexpected. Importantly, AI as a standalone tool helped overcome the low sensitivity of radiologists, and when used in conjunction with radiologists, it improved both specificity and AUC. These findings suggest not only an enhancement in AI performance but also a positive impact on clinical application and its interaction with doctors.

### Performance Interpretation and AI-Radiologist Interaction

In this study, Grad-CAM visualizations and prediction scores were provided simultaneously during the AI-assisted phase. Grad-CAM visualizations provided exploratory insight into the AI model’s decision-making process. In cases where the model predicted a higher probability of intussusception, the heatmaps tended to highlight bowel regions, particularly areas with mass-like soft tissue densities, abnormal bowel loop configurations, and adjacent bowel dilatation—radiographic features that are often associated with intussusception. Although this study was not designed to perform a detailed analysis of heatmaps in relation to the exact locations of intussusception, the findings suggest a hypothesis that the regions emphasized by the AI may correspond to the actual sites of intussusception. This observation warrants further investigation, as future studies could formally analyze whether AI-generated heatmaps could assist not only in detecting intussusception but also in localizing lesions more precisely, thereby expanding the clinical utility of AI models. Furthermore, although no formal usability or trust evaluations were conducted, future research will aim to systematically assess user experience and satisfaction with the AI platform. Although no single consistent pattern of false positives or false negatives was identified, image review revealed some recurring tendencies. For the AI model, false positives frequently occurred when bowel loops with visible gas were intermixed with adjacent loops lacking gas. False negatives were more likely in cases with diffusely reduced bowel gas, even outside the region of intussusception. For radiologists, false positives were often observed when gas was relatively sparse in the right upper quadrant. Regarding false negatives, both the AI model and radiologists missed cases, in which intussusception remained inconspicuous even on retrospective review, underscoring limitations inherent to the radiographic modality itself.

Interestingly, in this study, radiologist 1 had the least experience in both general and pediatric radiology, followed by radiologist 2 and radiologist 3. The use of AI had a more pronounced effect on improving the sensitivity and specificity of intussusception detection in doctors with less experience in pediatric radiology, such as general radiologists or clinicians, compared to pediatric radiology specialists. This finding is particularly significant, as it suggests that AI could be especially useful in emergency settings where pediatric radiologists are not always available around the clock. By aiding in the screening of patients using radiographs, AI can help identify patients who require further evaluation with ultrasonography or transfer to a specialized hospital for advanced diagnostic evaluation, supporting more effective decision-making in such environments.

### Strengths and Generalizability

Validation with other institutional datasets is challenging when developing AI algorithms. Many studies, not limited to pediatric radiology, develop their own AI algorithms and demonstrate high diagnostic performance. However, further validation with external datasets and real clinical adaptation is difficult, with one of the major reasons being the overfitting problem [32,33]. This challenge is more pronounced in pediatric radiology due to the relatively small dataset compared to adults, potentially leading to decreased diagnostic value when applying algorithms to other datasets. Nonetheless, attempting to demonstrate the diagnostic value itself is valuable for the safe adaptation of AI in clinical situations. While one study has performed external validation of AI on AXRs using a large dataset, validation through multicenter studies involving different hospitals and presenting the results is worthwhile [9]. In this study, to mitigate overfitting, we developed the AI model using a large dataset from 2 hospitals, expanding upon our previous work by incorporating high-volume data from hospital B. Additionally, the external validation results showed AUCs of 85.3% and 86.7% for hospital C and hospital D, respectively, as presented in Figure 3A, despite potential variations in imaging equipment and patient populations. These findings suggest that the AI model demonstrated consistent and generalizable performance across institutions. In this study, we aimed to include as many intussusception cases as possible from each participating institution. However, due to differences in hospital size, patient volume, and disease incidence, the absolute number of cases naturally varied across institutions. Data from larger hospitals were primarily used for retraining and upgrading the AI model, while data from smaller institutions were used for external validation through a reader study. Although the sample sizes were modest, incorporating multicenter data with diverse clinical environments introduced important variability, enhancing the clinical relevance of the model. Moreover, it is meaningful to consider the appropriate era for adopting AI in clinical practice. Screening young infants for intussusception is challenging due to poor cooperation and low incidence of typical triad symptoms. Consequently, frequent emergent ultrasonography is necessary, despite the actual incidence of the disease. However, delayed diagnosis could lead to worsening mechanical bowel obstruction, ischemia, pneumoperitoneum, and ultimately fatal problems. Therefore, assessing whether AI could be effectively used as one of the effective computer-assisted devices to triage and expedite ultrasonography scans is meaningful, as it demonstrates how AI could be applied in clinically necessary situations.

### Limitations and Future Directions

There are several limitations in this study. First, although this was a multicenter study, the number of patients in the external validation set was relatively small, reflecting the varying sizes of participating hospitals and the rarity of the disease. Nevertheless, the inclusion of multiple institutions with different characteristics could contribute to enhancing the generalizability of the algorithm. However, all hospitals were located within the same country; thus, differences in imaging equipment, patient demographics, and clinical protocols across international regions were not fully addressed. To overcome this, an international validation study based on the developed web-based platform is currently underway. Second, as a retrospective study, selection or spectrum bias and variability in data quality across centers may have been introduced. We attempted to mitigate these biases by applying consistent inclusion criteria, excluding radiographs with artifacts or external objects, and including all available intussusception cases with a fixed 3-4:1 ratio control group to reflect real-world prevalence without artificial matching. Although this led to an inherent class imbalance, we believe that maintaining the real-world prevalence ratio enhances the clinical applicability of the model. To mitigate potential bias from the imbalance, we used evaluation metrics such as AUC, sensitivity, and specificity, which are robust to class distribution differences. Furthermore, the external validation across multiple centers supports the generalizability of the model, despite the imbalance. Third, the control group was selected in reverse chronological order rather than by random sampling, which could introduce temporal sampling bias. Although quality control measures were applied across sites, this limitation could not be entirely avoided. Fourth, ROI annotation was performed by a single experienced pediatric radiologist without formal interrater reliability assessment. Although consistent anatomical landmarks were used to minimize variability, the potential for subjectivity remains. Fifth, only 3 radiologists participated in the reader study, which may limit the generalizability across different levels of experience. We included both trainees and a board-certified pediatric radiologist to reflect a range of expertise, but the limited number of readers remains a constraint. Moreover, during the AI-assisted phase, radiologists were exposed to the AI model’s prediction scores and Grad-CAM outputs, which could have introduced anchoring bias. However, this approach was intended to simulate real-world clinical practice, where AI outputs were used as supportive tools alongside radiologists’ interpretation. Compared to previous studies that only provided prediction scores, the inclusion of both visual explanations and scores was intended to enhance transparency and the interpretability of AI-assisted diagnosis.

In addition, the washout period could have contributed to a general improvement in the radiologists’ skills. Nevertheless, given that the diagnostic performance of radiographs remained limited even for experienced radiologists, this effect is unlikely to be a major confounding factor. Even if some skill improvement occurred, the results remain meaningful, as they demonstrate how AI assistance impacts diagnostic performance differently depending on the reader’s level of experience. Given that this study represents the first multicenter external validation following our initial single-center study, we considered it important to first conduct a reader study among radiologists, with varied levels of radiology experience, before expanding to performance evaluations involving broader clinical physician groups. Further prospective studies involving nonradiologist clinicians are planned as the next step to further validate the clinical utility of the AI model.

Given the clinical significance of applying AI in pediatric emergent diseases, further research using large datasets across diverse countries would be beneficial. Moreover, this study goes beyond research alone by presenting a user-applicable website that enables validation across diverse patient populations in different countries. While the current platform may benefit from further technical refinements for seamless clinical integration, its web-based accessibility provides a foundation for worldwide validation efforts and collaborative research. This aspect reinforces the study’s fundamental importance and its potential to serve as a cornerstone for future research.

### Conclusions

This study upgraded an AI model for the screening of ileocolic intussusception on pediatric AXRs and performed external validation using multicenter data. The upgraded AI model demonstrated improved diagnostic performance and effectively increased specificity and AUC, particularly benefiting radiologists with less experience in pediatric imaging. The AI model could serve as a supportive tool for screening and triage in emergency settings where pediatric radiologists are not always available. Furthermore, by presenting a user-applicable software platform, this study goes beyond theoretical research and facilitates broader validation across diverse patient populations and countries. Given its potential to aid in early detection and triage, further research with larger datasets and diverse clinical settings is warranted to support its clinical adoption.

## Supplementary material

10.2196/72097 Multimedia Appendix 1 Receiver operating characteristic (ROC) curve of the artificial intelligence (AI) model (dotted line) and individual reader performance on the external validation datasets (hospitals C and D). Bar plots on the right summarize the average sensitivity and specificity of readers with and without AI assistance.

10.2196/72097 Multimedia Appendix 2 Diagnostic performance of 5 cross-validated models on the internal test set and external validation cohorts.

## Abbreviations:

AI: artificial intelligence
AUC: area under the receiver operating characteristic curve
AXR: abdominal radiograph
Grad-CAM: Gradient-Weighted Class Activation Mapping
ROI: region of interest
STROBE: Strengthening the Reporting of Observational Studies in Epidemiology
