# Supplementary figures and images for: External Validation of an Upgraded AI Model for Screening Ileocolic Intussusception Using Pediatric Abdominal Radiographs: Multicenter Retrospective Study
Source: J Med Internet Res. 2025 Jul 8;27:e72097. doi: 10.2196/72097 (PMC12277635; doi:10.2196/72097)

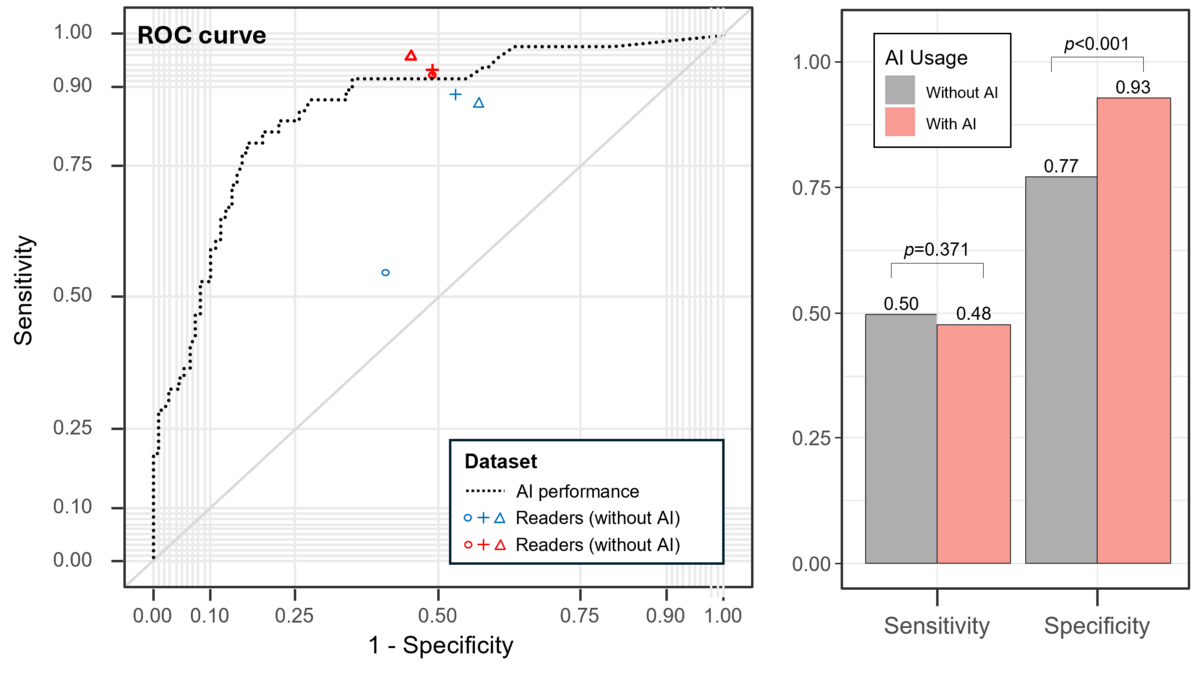

Supplement: Multimedia Appendix 1 [file jmir-v27-e72097-s001.png]
