# Supplementary material for: External Validation of an Upgraded AI Model for Screening Ileocolic Intussusception Using Pediatric Abdominal Radiographs: Multicenter Retrospective Study
Source: J Med Internet Res. 2025 Jul 8;27:e72097. doi: 10.2196/72097 (PMC12277635; doi:10.2196/72097)
Supplement: Multimedia Appendix 2 [file jmir-v27-e72097-s002.docx]

| **Fold** | Internal testset | External cohorts |
| --- | --- | --- |
| Fold 1 | 0.919 (0.884-0.954) | 0.850 (0.776-0.924) |
| Fold 2 | 0.933 (0.897-0.969) | 0.844 (0.789-0.899) |
| Fold 3 | 0.944 (0.913-0.976) | 0.826 (0.760-0.892) |
| Fold 4 | 0.932 (0.900-0.965) | 0.837 (0.769-0.905) |
| Fold 5 | 0.914 (0.870-0.959) | 0.856 (0.774-0.938) |
| Average | 0.928 (0.893-0.965) | 0.843 (0.774-0.912) |
